# Exploring the lipoproteome of Parageobacillus thermoglucosidasius

**DOI:** 10.1099/acmi.0.001170.v3

**Published:** 2026-04-10

**Authors:** Mingailė Jackson, Pawel Palmowski, Andrew Porter, Paul B.C. James, Iain C. Sutcliffe

**Affiliations:** 1 School of Geography & Natural Sciences, Faculty of Science and Environment, Northumbria University, Newcastle upon Tyne NE1 8ST, UK; 2 Newcastle University Protein and Proteome Analysis, Newcastle University, Newcastle upon Tyne NE2 4HH, UK

**Keywords:** ATP-binding cassette transporters, cell envelope, lipoprotein, *Parageobacillus*, sporulation

## Abstract

*Parageobacillus thermoglucosidasius* is a thermophilic endospore-forming *Bacillales* of considerable biotechnological interest. Bacterial lipoproteins are a significant class of cell envelope components, influencing multiple aspects of the interactions of bacteria with their environments. We have therefore used a bioinformatic approach to identify the lipoproteins encoded in the *P. thermoglucosidasius* DSM 2542 type strain genome. Eighty-nine putative lipoproteins were found, representing ~2.3% of the *P. thermoglucosidasius* reference proteome; 84% (75) of these were also found in the predicted proteome of *P. thermoglucosidasius* strain Y4.1MC1. Just over half of the 89 putative lipoproteins are predicted to be substrate-binding proteins in ATP-binding cassette importer systems; others function in signalling pathways, protein translocation, redox processes, including the respiratory chain, and as enzymes. At least 10 lipoproteins are predicted to be involved in *P. thermoglucosidasius* spore cycle, whilst 15 are lipoproteins of unknown function. Proteomic analysis of *P. thermoglucosidasius* grown to exponential phase in Lysogeny Broth medium detected the expression of nearly 60% (52 out of 89) of the predicted lipoproteome, with the notable exception of those functionally linked to sporulation. These data contribute to the understanding of the *P. thermoglucosidasius* cell envelope and so should be of use to those studying the physiology, metabolism and biotechnological utility of this bacterium.

**Impact Statement**

The cell envelope is of importance as the interface between a bacterium and its environment. Bacterial lipoproteins, which are tethered within the cell envelope, carry out diverse functions. To further understand the biology of the biotechnologically important bacterium *Parageobacillus thermoglucosidasius*, we have used computational tools (bioinformatics) to identify lipoproteins encoded in the genome of the type strain of this species. In addition, we have used proteomics to provide an initial analysis of which lipoproteins are expressed by *P. thermoglucosidasius* during growth under standard laboratory conditions. These data presented will provide a resource of use to those working with this bacterium and its relatives, particularly in the context of cell envelope interactions.

## Data Summary

The authors confirm that all supporting data and protocols have been provided within the article or through supplementary data files. Six supplementary data files are uploaded to Microbiology Society Figshare (https://doi.org/10.6084/m9.figshare.31557805)[1] containing (1) lipoprotein signal peptide identification, (2) proteomic methods, (3) analyses of lipoprotein signal peptide features, (4) summary data on lipoprotein functional predictions, (5) expanded information on lipoprotein functional predictions and (6) data from proteomic detection of lipoproteins and other proteins.

The mass spectrometry raw data are available at MassIVE, Centre for Computational Mass Spectrometry (https://massive.ucsd.edu/ProteoSAFe/static/massive.jsp?redirect=auth), under Submission ID MSV000100471.

## Introduction

Bacterial lipoproteins (BLPs) are peripheral membrane proteins that are localized by post-translational modification with a diacylglycerol anchor via a biosynthetic pathway that is unique to bacteria [2,5]. BLPs are universally present in bacteria [2,6] and have diverse and versatile functions. In diderm *Pseudomonadota*, BLPs typically play essential roles in outer membrane assembly and function [7], whereas their functions in monoderm (Gram-positive) bacteria are often analogous to periplasmic proteins of diderm bacteria [3,4]. The defining feature of BLP anchoring is thioether modification of a signal peptide cysteine by diacylglycerol [2,3, 5, 7]. This critical cysteine is part of a conserved signal peptide motif known as the ‘lipobox’. Following preprolipoprotein translocation to the plasma membrane surface by Sec or TAT, the lipid modification is carried out by prolipoprotein diacylglyceryl transferase (Lgt), followed by signal peptide cleavage proximal to the lipidated cysteine by a dedicated lipoprotein signal peptidase II (Lsp) [5,7]. Following signal peptide cleavage, the newly exposed amino-terminal cysteine can be further modified with an amide-linked fatty acid by lipoprotein N-acyltransferase (Lnt) or alternative enzymes [5,8], although this step is not universally conserved; triacylation of BLP is typically associated with trafficking of BLP to the outer membranes of diderm bacteria [5,7]. The conserved lipobox features of BLP as part of distinctive ‘type II’ signal peptides have greatly facilitated their cataloguing in genomes using bioinformatic tools [9,13], which has in turn allowed recognition of their functional diversity [3,4].

*Parageobacillus thermoglucosidasius*, formerly classified as *Geobacillus thermoglucosidasius* [14], is a thermophile of biotechnological interest, both as a novel source of enzymes and for whole-cell biocatalysis, for which the molecular toolkits for working with strains are expanding [15,18]. Usefully, a wide range of substrates, including lignocelluloses, can be fed into bioethanol and other biofuel production [15,16]. Some strains, including the type strain DSM 2542, have been shown to carry out carbon monoxide-mediated hydrogen production via a carbon monoxide dehydrogenase/energy-converting hydrogenase complex (CO+H_2_O → CO_2_+H_2_) [19]. The species is also of interest as a representative of the thermophilic endospore-forming *Bacillales*.

To support a better understanding of the biology of *P. thermoglucosidasius*, we have conducted a bioinformatic analysis of the lipoproteome of the type strain, DSM 2542, and further used proteomics to examine BLP expression in this strain.

## Methods

### Proteomes

The *P. thermoglucosidasius* DSM 2542 type strain proteome UP000033360 (3,652 proteins), derived from GenBank CP012712.1 [20], was downloaded from UniProt proteomes. Subsequently, the proteome was cross-referenced with the UniProt reference proteome UP000093052 (3,686 proteins) derived from GenBank CP016622.1 [21] for strain NCIMB 11955, an alternative deposit of the type strain, which helped resolve minor annotation differences. The *P. thermoglucosidasius* strain Y4.1MC1 proteome was downloaded from UniProtKB (3603 proteins), derived from GenBank CP002293.

### Lipoprotein pattern searching and dataset validation

Putative BLPs were identified using the ScanProsite tool [22] (https://prosite.expasy.org/scanprosite/) to search the selected proteomes with BLP-specific sequence motifs. Searches were conducted using the G+LPPv3 motif ‘<[MV]-X(0,13)-[RK]-(1-4)(6,20)-[LIVMFESTAGPCW]-[LVIAMFTG]-[FIVMSTAGCP]-[AGS]-C’. This is an updated version of the G+LPPv2 motif described in Rahman *et al*. [11] which additionally permits amino acids F at −2 and W at −5 relative to the lipobox cysteine. Further searches were conducted using the Prosite motif PS51257 defined for BLP. To explore whether some sequences had extended signal peptide N-regions, the G+LPPv3 search was also repeated with the start features amended to ‘<[MV]-X(0,25)-[RK]-…’ (G+LPPlong motif).

To validate the BLP dataset, hits recovered from pattern searching were collated and analysed using a consensus approach to signal peptide prediction [11], with sequences analysed using SignalP 3.0 (https://services.healthtech.dtu.dk/services/SignalP-3.0/ [23]) and Phobius (https://phobius.sbc.su.se/ [24]) to analyse N- and H-region features, combined with BLP prediction using SignalP 6.0 (https://services.healthtech.dtu.dk/services/SignalP-6.0/ [25]), LipoP 1.0 (https://services.healthtech.dtu.dk/services/LipoP-1.0/ [12]) and PRED-LIPO (http://bioinformatics.biol.uoa.gr/PRED-LIPO/input.jsp [13]). Hits were considered to be false positives where the majority of tools reported discrepant results, whilst the features of a small number of sequences were designated as unclear (File S1, available in the online Supplementary Material). Some ambiguous sequences were further checked using TOPCONS (https://topcons.cbr.su.se/pred/ [26]) and DEEPTMHMM (https://dtu.biolib.com/DeepTMHMM [27]) to help distinguish signal peptide H-regions from transmembrane helices. Signal peptide features of BLP were visualized using WebLogo (https://weblogo.berkeley.edu/ [28]).

### BLP functional analyses

BLP sequences were obtained via UniProt (https://www.uniprot.org/ [29]). Homology transfer analysis was performed using the NCBI BlastP suite (https://blast.ncbi.nlm.nih.gov/Blast.cgi [30]), first searching for homologues in the Protein Data Bank (PDB) dataset; where homologues weren’t identified in PDB, searches were repeated against the reference proteins (refseq) dataset. Conserved domains detected by blastp analysis were cross-referenced with those annotated in UniProt, notably InterPro and Pfam entries [31]. Chromosomal context was explored via the KEGG gene cluster and genome browser annotation [32] for *P. thermoglucosidasius* DSM 2542, accessed via the UniProt entries for each BLP. BLP homologues in *Bacillus subtilis* were identified by tblastn searches against the *B. subtilis* strain 168 reference genome.

### Proteomic analysis

Overnight cultures of *P. thermoglucosidasius* DSM 2542 grown in LB medium (with additives of 1.05 mM nitrilotriacetic acid, 0.59 mM MgSO_4_, 0.04 mM FeSO_4_ and 0.91 mM CaCl_2_) at 55°C, shaking at 200 r.p.m., were inoculated into 10 ml volumes of fresh medium at a starting OD of 0.05 and grown to exponential phase (~5 h). Cultures were centrifuged at 4,000 ***g*** for 10 min, and supernatants were collected for Triton X-114 extraction using the method of Cockayne *et al*. [33].

Triplicate samples were processed for proteomic analysis. Briefly, proteins were trypsin-digested in S-Trap micro cartridges. Five microlitres of the resulting peptide solution were analysed using a Thermo UltiMate 3000 RSLCnano LC-MS in DIA mode. Peptides were separated on a 75 cm, 2 µm particle EasySpray column over a 60 min non-linear gradient of acetonitrile (to 35%) and injected online into a Q Exactive HF mass spectrometer. Data were searched with DIANN software against the *P. thermoglucosidasius* proteomes to identify peptides and proteins in the samples. Detailed methods are given in File S2.

## Results and discussion

### BLP biosynthesis pathway in *P. thermoglucosidasius*

The key BLP biosynthetic enzymes Lgt and Lsp are present as single copies in the *P. thermoglucosidasius* type strain genome (PF01790 Lgt, AOT13_00885; PF01252 Lsp, AOT13_08700). As in other members of *Bacillota*, Lnt, which adds an amide-linked third fatty acid, is absent. Moreover, homologues of the alternative amino-acylation system LnsA/LnsB of *Staphylococcus aureus* [34] are also absent, suggesting that only diacyl-BLPs are produced by this species. In addition, although various members of *Bacillota* are able to generate ‘lyso-form’ BLP by migrating a fatty acid from the diacylglycerol onto the N-terminal cysteine using the enzyme lipoprotein intramolecular transacylase [35], no homologues of *Bacillus cereus* Lit (PF07314, BC_1526) are present in the *P. thermoglucosidasius* type strain genome. Some members of *Bacilllota*, including *Geobacillus kaustophilus*, are noted to synthesize N-acetyl BLP [36]. N-acetylation in *B. subtilis* requires synthesis of an acetylated heptaprenylglyceryl carrier from which the acetyl group is then transferred to BLP by a transferase (YpjA, renamed LhaT [37]). Homologues of the key enzymes in this pathway (notably PcrB and YvoF for carrier synthesis; LhaT, AOT13_15495) are all encoded in the *P. thermoglucosidasius* type strain genome, and YvoF (AOT13_00870) appears to be in an operon with Lgt, as in *B. subtilis*. A phosphatase (YvoE, AOT13_00875) may also participate in carrier synthesis. Thus, it seems likely that some BLPs in *P. thermoglucosidasius* are N-acetylated.

### Signal peptide features of the BLP dataset

Pattern searches identified 95 potential BLPs in the *P. thermoglucosidasius* type strain proteomes, of which signal peptide feature analysis confirmed 86 predicted BLPs, 3 unclear and 6 false-positive sequences (File S1). Hence, BLPs represent ~2.3% of the *P. thermoglucosidasius* reference proteome (3,686 proteins), comparable to other members of *Bacillota* [4,9, 10, 13, 38]. Seventy-five of these BLPs, including the 3 unclear sequences, with identical [39] or near-identical [38] sequences, were also found in the proteome of *P. thermoglucosidasius* strain Y4.1MC1. Conversely, nine BLPs were identified in the proteome of strain Y4.1MC1 that were absent from the *P. thermoglucosidasius* type strain proteome, including four sequences that are annotated as pseudogenes in the latter (File S1; see below). None of the BLPs were predicted by SignalP 6.0 to be TAT substrates, suggesting that all BLPs are delivered to Lgt by the Sec translocase. Indeed, inspection of the BLP signal peptides confirmed that only 3 out of 89 contained an RR ‘twin arginine’ sequence, in each case in non-canonical motifs.

The mean lipobox cysteine number, reflecting the signal peptide length, was 21.4±2.7 lipobox (with 79% in the range 19–23; File S3, Table S1 and Fig. S1) consistent with previous analyses of BLP from Gram-positive bacteria [10,13, 38, 39], as these signal peptides are typically shorter than those of secretory proteins. Reflecting this, the mean Type 1 (Sec) signal peptide cleavage site probability for these sequences was low (mean 0.620±0.224, only 13 of 86 sequences >0.8; File S3, Table S1, Fig. S2), as observed previously for BLP from Gram-positive bacteria [11].

**Fig. 1.**
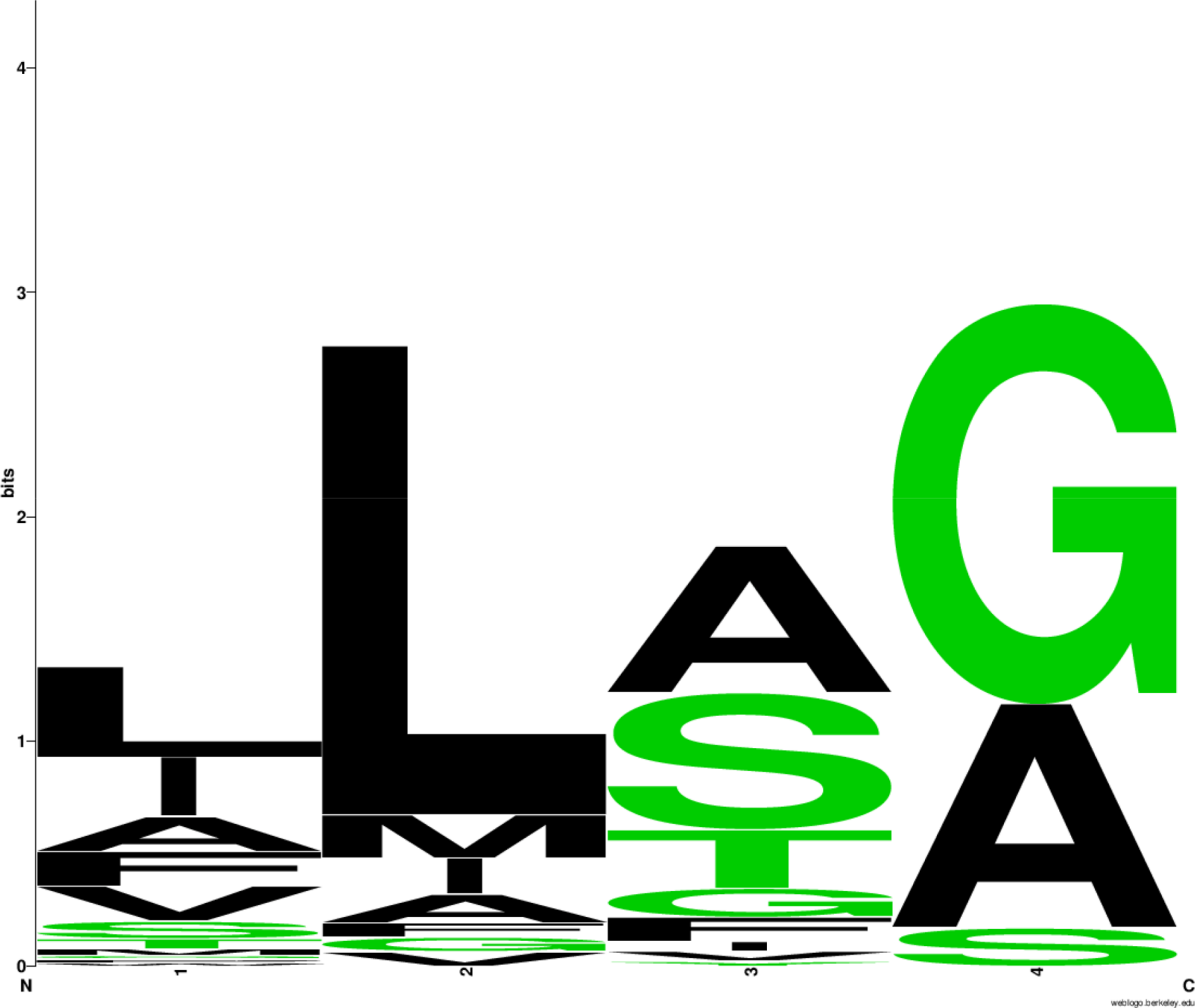
Lipobox sequence conservation in 86 *P*. *thermoglucosidasius* BLPs.

**Fig. 2.**
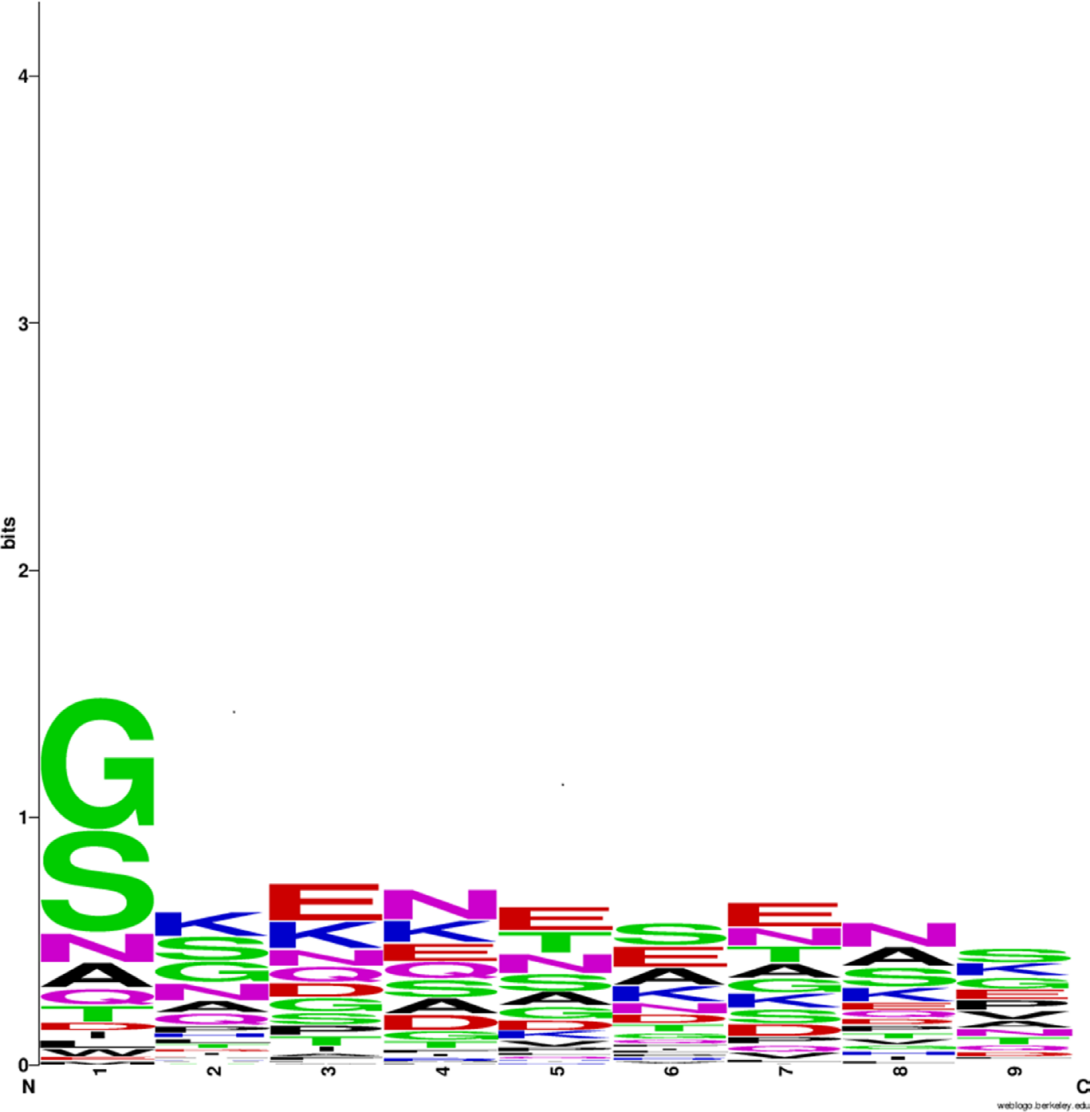
N-terminal features of 86 *P*. *thermoglucosidasius* BLPs from the +2 position adjacent to the lipidated cysteine to amino acid 10 of the mature BLP.

The lipobox features (Fig. 1, File S3, Table S2) showed a strong preference for leucine at the −3 position (76%); alanine, serine and threonine at −2 (82%); and alanine or glycine at −1 (94%). These lipobox features are consistent with those previously reported for BLP from other *Bacillota* and monoderm bacteria [10,13, 38, 40]. Sequence conservation is also notable at the +2 position relative to the lipobox cysteine, with a preference for glycine (36% of sequences) and serine (28%) (File S3, Table S3). Overall, glycine, serine, alanine and asparagine are present in 79% of sequences at this position (Fig. 2), consistent with previous reports for BLP from other *Bacillota* and monoderm bacteria [10,13, 41]. Kumari *et al*. [41] reported that aspartate at +2 helps anchor BLP to the plasma membrane, likely reflecting interaction of the negative amino acid side chain with positively charged phospholipid head groups (notably lysyl-phosphatidylglycerol in *S. aureus*). Although *P. thermoglucosidasius* has amino-group-containing lipids in its cell membrane, including abundant phosphatidylethanolamine [42], it is notable that aspartate and glutamate are rare in the +2 and +3 positions of the mature termini of the BLP (3.5% of sequences). In contrast, lysine is common at +3 (16%; File S3, Table S3, Fig. S2).

Serine, glycine, threonine and asparagine have been noted to be common in the +2 to +7 positions of the mature BLP sequences [13]. Here, these amino acids were found to make up 43% of the amino acids in the +2 to +10 positions of the N-termini of the mature BLP (Fig. 2; File S3, Table S3). In addition, these N-termini typically had an overall net negative charge (*n*=46, 53.5% of sequences) or were net neutral (28%), reflecting a predominance of aspartate and glutamate over lysine, notably in the +4 to +10 positions (File S3, Tables S3 and S4; Fig.S2). Strikingly, bulky amino acids (phenylalanine, tryptophan, tyrosine and histidine) were notably absent in these positions (File S3, Table S3).

Based on visual assessment of 70 BLPs in strain Y4.1MC1 for which AlphaFold-predicted structures are available in UniProt, it was noted that, as reported previously for Sec signal peptides [43], the BLP signal peptide sequences typically appear well separated from the main folded part of the proteins. Moreover, it was noted that most of these BLPs have a flexible ‘linker’ observable in the N-terminal region of the mature BLP. These N-terminal linker sequences, distinct from the main body of the mature BLP, have a mean length of 17.1±11.0 amino acids (File S3, Fig. S3; range 2–71 amino acids, 80%>8) and presumably confer flexibility for protein interactions at the membrane surface.

### BLP functions in *P. thermoglucosidasius*

Functional predictions for the 86 predicted BLP and 3 unclear sequences are given in Files S4 and S5.

### BLP as substrate-binding proteins in ABC transport systems

As in other monoderm bacteria [3,4, 44], substrate-binding proteins (SBPs) in ATP-binding cassette importer systems were the most abundant group of BLP (45 out of 89, 51%). SBP interacts with integral membrane permeases to help confer substrate specificity and directionality (import) to transport. Protein family and domain analysis allowed prediction of substrates for these SBPs (Table 1; File S4). However, these should be considered as broad functional classes, as precise homology-based substrate prediction for SBP is challenging (for example, see [45]). The substrate profile overall indicated a preponderance of ABC transporters for amino acids/peptides (18 out of 45) and sugars/carbohydrates (11 out of 45), along with various ions (13 out of 45). Examination of the chromosomal context of each SBP allowed these systems to be categorized (Table 1) as complete (SBP, permease and ATPase components present) or incomplete (SBP and permease only), whilst two are ‘orphans’ (no proximal permease and ATPase components). The nine incomplete systems are likely completed by interaction with promiscuous ATPase components from related ABC transport systems encoded elsewhere in the genome, as observed in other bacteria [44,46]. Multitask ATPase components have previously been typically associated with sugar importers, as is also the majority here (six incomplete systems), whilst in three cases, other substrates are involved. For the AOT13_05010 cystine SBP, this system is incomplete as the cognate ATPase is a pseudogene in the type strain genome. However, this incomplete ABC system is in a chromosomal locus associated with a complete ABC transport system containing two MetQ family methionine-binding SBPs (AOT13_05040, AOT13_05055). Hence, the functionality of these systems may be inter-related. The phosphate-binding SBP AOT13_16790 and associated permeases are likely completed by the AOT13_16750 ABC phosphate transporter ATPase. Intriguingly, the two orphan SBPs are both predicted to bind metal ions and may interact with ABC permease/ATPase components encoded elsewhere in the genome (Tables S4 and S5). Also of note is that the peptide/nickel transport system SBP AOT13_04975 is 1 of the 20 proteins noted to be encoded in 2 genetic loci identified as unique to hydrogenogenic strains [47], presumptively because both the carbon monoxide dehydrogenase and the associated energy-converting hydrogenase complex require nickel as a cofactor [19].

**Table 1.** Substrate groups for SBP of ATP-binding cassette transport systems

Pfam family	Substrate group	N	Complete/incomplete	Detected in proteome
PF00496	Peptides and related	6	6/0	3/6
PF00497	Amino acids	4	3/1	4/4
PF03180	MetQ/NlpA family, methionine binding	4	4/0	4/4
PF04392	Aromatic amino acids (Y, F, W)	2	2/0	2/2
PF13458	Aromatic/aliphatic compounds and branched-chain amino acids	2	2/0	1/2
PF04069	Taurine	1	1/0	1/1
PF01547	Sugars/oligosaccharides	5	1/4	2/5
PF13407	Sugars	3	3/0	1/3
PF13416	Sugar phosphates, alditol phosphates	3	2/1	2/3
PF13426	Sugars/oligosaccharides	1	0/1	1/1
PF02608	Nucleosides (Bmp family)	1	1/0	1/1
PF09084	Nitrate/sulphonate/bicarbonate (NMT1/THI5-like)	3	3/0	1/3
PF13379	Nitrate/sulphonate/bicarbonate (NMT1-like)	2	2/0	0/2
PF12849	Phosphate	1	1/0	1/1
PF01497	Iron-siderophore and related	5	4/1	5/5
PF01297	Zn/Mn	1	Orphan	1/1
PF13531	Molybdenum	1	Orphan	1/1

The sugar-binding SBP AOT13_11435 shows significant identity to *Agrobacterium tumefaciens* ChvE [48] and is part of a complete ABC transport system. As well as participating in sugar (arabinose, galactose) uptake by *A. tumefaciens*, sugar-bound ChvE interacts with the VirA/VirG two-component signalling system (TCS) via interaction with the extracytoplasmic domain of VirA, thereby also influencing gene expression [49]. It is noted that the AOT13_11435 locus also contains genes encoding a YesM-YesN TCS (AOT13_11425, AOT13_11430), raising the possibility of similar interactions and/or the regulation of the ABC transport system by the TCS. Alternatively, a second non-BLP putative secreted arabinose binding protein (AOT13_11420, PF13407) encoded in this locus may interact with the TCS; the wider genomic context encodes multiple proteins linked to arabinose catabolism and the incomplete ABC sugar transport system containing SBP AOT13_11475.

Similarly to AOT13_11435, it was noted that two other SBP genes are located adjacent to TCS. The incomplete ABC locus containing SBP AOT13_11530 is also located adjacent to a YesM-YesN TCS. AOT13_11530 is homologous to the XBP1 xylo-oligosaccharide binding protein of *Caldanaerobius polysaccharolyticus* (PDB 4G68_C) [50]. Both these SBPs are part of gene clusters (11 genes in *P. thermoglucosidasius*) encoding TCS, xylosidases and enzymes for xylose metabolism. Hence, the cognate TCS may regulate expression of this gene cluster, not least as an additional accessory protein comparable to XylFII in *Clostridium beijerinckii* (see below) is not present. The AOT13_11115 glutamate/amino acid SBP, part of a complete ABC system, is also encoded in a locus with a GlnK-GlnL TCS which may regulate its expression, as has been reported in *Streptomyces coelicolor* [51].

### Other possible BLP interactions with TCS

AOT13_14265 is a BLP with unclear signal peptide features and a predicted seven-bladed beta-propeller fold that is likely encoded in an operon with a TCS. As with the interaction of ChvE and VirA/VirG [48] noted above, it is apparent that many TCSs interact with an extracytoplasmic accessory protein as ‘three-component systems’, including the xylose-sensing XylFII-TCS interaction in *C. beijerinckii* [52]. Most notably, the actinobacterial beta-propeller domain protein LpqB interacts with MtrAB TCS to regulate multiple cell envelope functions [4,53]. Therefore, it seems likely that AOT13_14265 interacts with the TCS histidine kinase AOT13_14255.

AOT13_04650 is a BLP belonging to the Spa1 family of lantibiotic immunity lipoproteins [54,55], exhibiting 82 out of 144 (57%) amino acid identities to the GeoI Spa1 family protein of *Geobacillus thermodenitrificans* [56]. As in other lantibiotic immunity systems, this protein is encoded in a locus that also encodes ABC permease and ATP-binding protein components for lantibiotic export (SpaGEF [54,57]) and adjacent to a SpaKR/NisRK TCS (Table 2). Spa1 immunity BLP binds lantibiotics, although the precise mechanisms conferring immunity are unclear [54]. The role of the associated TCS is also unclear, as it is notable that, in contrast to related systems, including the geobacillin I biosynthetic gene cluster of *G. thermodenitrificans* [56], the *P. thermoglucosidasius* gene cluster lacks genes for a lantibiotic precursor and for lantibiotic maturation (Table 2), as previously reported for multiple *Parageobacillus* strains representing *P. thermoglucosidasius* (including DSM 2542/NCIMB 11955) and *Parageobacillus toebii* [15]. Intriguingly, GY4MC1_2037 is annotated as a lantibiotic in the *P. thermoglucosidasius* strain Y4.1MC1 genome, and an identical 41-amino-acid sequence is encoded but not annotated in the type strain genomes, adjacent to the AOT13_12155 SCO1 BLP (see below). Although an l-threonine dehydratase, AOT13_12145, is encoded nearby, other genes encoding lantibiotic maturation and export systems are not evident, and the sequence lacks homology to known lantibiotics and so may be misannotated. Thus, it remains unclear if AOT13_04650 will contribute to self-immunity or immunity to exogenous lantibiotics.

**Table 2.** Comparison of the proteins encoded in the *G. thermodenitrificans* geobacillin I gene cluster with the lantibiotic resistance clusters of *P. thermoglucosidasius* strains

	*G. thermodenitrificans* NG80-2	Name/function	*P. thermoglucosidasius*	Protein identity	Y4.1MC1
Unrelated	GTNG_0263	Aspartyl/glutamyl-tRNA amido-transferase subunit B	AOT13_04940	463/476 (97%)	GY4MC1_3491
Unrelated	GTNG_0264	O-acetylhomoserine sulfhydrylase	AOT13_05000	368/431 (85%)	GY4MC1_3484
GeoAI	GTNG_0265	SunA lantibiotic precursor	No hit, e=10		Absent
GeoB	GTNG_0266	SpaB dehydratase	No hit, e=0.05		No hit
GeoT1	GTNG_0267	SpaT ABC transporter (fused permease/ATPase)	AOT13_04810	158/591 (27%)	No hit
GeoC	GTNG_0268	SpaC LanC cyclase	No hit, e=0.05		No hit
GeoR	GTNG_0269	SpaR subtilin biosynthesis regulatory protein (TCS)	AOT13_04670	194/231 (84%)	GY4MC1_3546
GeoK	GTNG_0270	SpaK histidine kinase (TCS)	AOT13_04675	316/473 (67%)	GY4MC1_3545
GeoI	GTNG_0271	SpaI BLB	AOT13_04650	82/144 (57%)	GY4MC1_3550
GeoG	GTNG_0272	SpaG ABC permease	AOT13_04655	128/204 (63%)	GY4MC1_3549
GeoE	GTNG_0273	SpaE ABC permease	AOT13_04660	171/247 (69%)	GY4MC1_3548
GeoF	GTNG_0274	SpaF putative ABC transporter ATP-binding protein	AOT13_04665	183/229 (80%)	GY4MC1_3547
Unrelated	GTNG_0275	Glycine betaine/l-proline ABC transporter (permease), putative	AOT13_01690	36/145 (25%)	GY4MC1_0241
Unrelated	GTNG_0276	Quaternary amine transport ATP-binding protein	AOT13_13310	106/237 (45%)	GY4MC1_1826

AOT13_04795 is annotated as a CamS family sex pheromone protein and exhibits ~33% full-length sequence identity with the *S. aureus* CamS BLP. The signal peptide h-region of *S. aureus* CamS is notable for encoding the cAM373_SA hexapeptide that is released by an Eep intramembrane protease and acts as a pheromone signal potentiating conjugation [58]. However, the h-region hexapeptide of AOT13_04795 (LLLFLSS) is only one amino acid identical to cAM373_SA (AIFILAA), and so a similar signalling role in *P. thermoglucosidasius* cannot be confidently predicted. Notably, the mature BLP sequence of *S. aureus* CamS has been implicated in the downregulation of cytotoxin production, likely through interacting as a three-component system with the SaeRS TCS [59]. As for *S. aureus* CamS, the genomic locus encoding AOT13_04795 encodes a DNA ligase and a DNA helicase, but no TCS is present. It is thus possible that AOT13_04795 interacts with a TCS encoded elsewhere in the genome.

### BLP involved in protein translocation

As in other *Bacillota* [10,60, 61], two paralogous BLP members of the YidC family were identified, AOT13_03085 and AOT13_11805 (51% amino acid identity). Members of the YidC family are insertases facilitating the insertion of integral membrane proteins into the plasma membrane. As in homologous proteins, these BLPs have five membrane-spanning domains in addition to their predicted N-terminal lipid anchor. The substrates for these insertases are unknown, but in other *Bacillota*, the YidC paralogues exhibit both some functional redundancy and also some functional distinctiveness [60,62]. In *B. subtilis*, YidC (SpoIIIJ, ~70% amino acid identity with AOT13_03085) has an essential role in sporulation (see below), whilst YqjG influences competence [60]. As in *B. subtilis* and other *Bacillota*, AOT13_03085 is encoded in an operon with an ssDNA/RNA binding regulatory protein (KhpB/Jag/EloR); in streptococci, Jag/EloR and YidC appear to functionally interact [62,63].

A third BLP likely involved in protein translocation and folding is the predicted foldase AOT13_06770 (PrsA). Initially characterized in *B. subtilis*, these diverse peptidyl-prolyl isomerase family proteins are widespread in the *Bacillota*, where they likely assist folding of a range of translocated substrates [64,66], a function presumably enhanced by the location as BLP at the membrane-extracytoplasmic interface.

Cumulatively, these three proteins are likely to affect the correct localization, folding and functionality of a range of proteins translocated by *P. thermoglucosidasius*.

### Enzymes

Three BLPs are predicted to be enzymes. Consistent with their location at the membrane-wall interface, AOT13_14005 and AOT13_15985 are predicted carboxypeptidases acting on peptidoglycan stem peptides. AOT13_15985 is homologous to the *B. subtilis* DacB dd-carboxypeptidase which impacts on peptidoglycan cross-linking during sporulation [67]. As in *B. subtilis*, this protein is encoded in a locus with SpmA and SpmB GATE-domain membrane proteins that impact on spore formation. AOT13_14005 is a predicted ld-carboxypeptidase homologous to *B. subtilis* LdcB (YodJ) BLP, which is involved in peptidoglycan stem tetrapeptide remodelling, likely to allow d-ala recycling [68].

AOT13_13280 is a predicted quinoprotein sugar dehydrogenase, homologous to other secreted/periplasmic dehydrogenase enzymes [69]. The homologues of this protein require a pyrroloquinoline quinone cofactor. As the pyrroloquinoline quinone biosynthetic pathway, including the PqqA precursor [70], appears to be absent in *Parageobacillus*, it is likely that this cofactor needs to be scavenged from environmental sources.

### BLP with redox and related functions

The location of BLP in proximity to the membrane surface means they are well suited to roles in electron transfer [3]. Six BLPs are predicted to participate in the *P. thermoglucosidasius* respiratory chain. As in *Bacillus*, the respiratory chain has two terminal branches, with electrons shuttled from reduced menaquinone MK7 to distinct terminal electron acceptors, including cytochrome bd, cytochrome aa3 menaquinone oxidase (Qox) and cytochrome caa3 [71,72]. AOT13_08420 is the cytochrome c oxidase caa3 subunit II (CtaC), with two additional membrane-spanning domains, as in *B. subtilis* and other *Bacillales* [36,73]. Likewise, BLP AOT13_03340 is the subunit II component (QoxA) of the terminal quinol oxidase aa3, which also has two additional membrane-spanning domains. It remains unclear why the topology of these two integral membrane proteins benefits from the N-terminal BLP anchor. AOT13_00985 is 82 out of 111 (74%) amino acids identical to the lipoprotein cytochrome C-551 of thermophilic *Bacillus* sp. PS3 [74] and likely transfers electrons to the CbaABD cytochrome bo_3_-type cytochrome c oxidase [75], although it may alternatively shuttle electrons between cytochrome bc complex and cytochrome c oxidase caa3 [71]. In addition to these BLPs that are structural components of the respiratory chain, two BLPs likely participate in cytochrome c assembly: AOT13_12155 (SCO1/SenC) is homologous to *B. subtilis* YpmQ, whilst AOT13_08005 is a YtkA-like domain-containing protein. YtkA (CtaK) and YpmQ appear to be involved in the delivery of copper to the CuA copper centre in cytochrome caa3 [76]. Finally, AOT13_00095 is a Cu-Zn type superoxide dismutase. In mycobacteria, a Cu-Zn superoxide dismutase BLP (SodC) is part of the cytochrome c bc–aa3 (III–IV) respiratory chain supercomplex, where it may protect against superoxide radicals generated as a byproduct [77,78], and so a similar function might be envisaged for AOT13_00095.

Four other BLPs likely have redox-related activities. AOT13_07200 and AOT13_12855 are putative multicopper oxidases. AOT13_12855 (which is absent from strain Y4.1MC1) is a predicted laccase and exhibits 1 out of 529 (95%) sequence identity to *Geobacillus yumthangensis* pLacGy laccase, which has been characterized as degrading a range of phenolic substrates [79]. AOT13_07200 shows high (~80%) amino acid identity with *Geobacillus* copper-containing nitrite reductases [80]. The activity of this protein may be connected to another BLP, AOT13_14630 (NosL). In *Pseudomonas stutzeri*, NosL is a BLP that relays copper to periplasmic NosD in complex with the NosFY ABC permease/ATPase system [81]. NosDFY delivers copper to the NosZ nitrous oxide reductase, and a comparable gene cluster is present in *G. thermodenitrificans* in which NosL is replaced by a YtkA-domain containing copper delivery protein [82]. Intriguingly, AOT13_14630/NosL is part of a locus (AOT13_14620–AOT13_14640) encoding NosDFY but which lacks the NosZ nitrous oxide reductase. Hence, this putative relay system may deliver copper to an alternative recipient, with the AOT13_07200 copper-containing nitrite reductase as a likely candidate: it is noted that a homologue of this protein in *G. stearothermophilus* (B4109_3053, 77% identity) is encoded in a gene cluster encoding NosL, NosD and an ABC permease/ATPase. An alternative candidate may be BLP AOT13_18635 which has a C-terminal cytochrome c-like peroxidase domain and an N-terminal seven-bladed beta-propeller domain; the latter matches the IPR011045 copper-containing domain found in nitrous oxide reductases.

### BLP involved in the sporulation cycle

Involvement of BLP in the *Bacillus* endosporulation cycle has long been established, as ‘GerF’ germination mutations, which result in failure to respond to several germinants, were localized to *lgt* in *B. subtilis*, and several germination receptors are predicted BLPs [83]. In addition to AOT13_03085 (YidC) and AOT13_15985 (DacB) considered above, ten BLPs are predicted to be involved in the *P. thermoglucosidasius* spore cycle, notably in relation to the germination stage.

Three GerX(C) family BLPs are components of germinant receptor complexes. These are trimers of a GerX(C) BLP and two integral membrane proteins located in the spore inner membrane [84]. Gao *et al*. [85] have recently shown that in *B. subtilis*, these likely form a pentamer of trimers that function as ion channels opening in response to germinants such as l-alanine, although the role of the BLP remains unclear. Each of the GerX(C) BLP (AOT13_13755, AOT13_13830 and AOT13_15685) is encoded in loci with cognate GerA and GerB integral membrane proteins. It may be speculated that the BLP acts as an initial receptor, delivering germinants to the GerAB membrane complex, analogous to SBP. In addition, AOT13_02210 is a GerD family BLP, with 75% identity to the GerD protein of *G. kaustophilus* [86]. GerD proteins are proposed to act as a scaffold clustering germinant receptors in a spore inner membrane supercomplex termed the ‘germinosome’ [86,87]. The precise nature of the germinants interacting with these receptors remains unknown, but various sugars (notably glucose) and/or amino acid mixtures can stimulate germination of *P. thermoglucosidasius* DSM 2542 spores [88,89].

Four additional BLPs belong to the PF09580 YhcN/YlaJ spore lipoprotein family. In *B. subtilis*, these have been found to influence spore plasma membrane fluidity and thereby likely stabilize membrane complexes (such as the germinosome), ensuring efficient germination responses [90]. YhcN and other proteins in this family are noted to have a ring-building motif that likely directs protein oligomerization, although ring formation by isolated YhcN could not be observed *in vitro* [91]. As in several other *Bacillales*, including *B. cereus*, although not *B. subtilis*, one member of this family, AOT13_07505, is encoded in a locus along with a spoVAC-spoVAE operon flanked by PF07870 (DUF1657) proteins and a ‘2DUF’ protein [92]. This locus likely contributes to the release of spore calcium-dipicolinic acid, one of the key early stages of germination [84]. In *B. cereus*, YlaJ (47% identity to BLP AOT13_08380) is predicted to form a trimeric complex with the spore cortex lytic enzyme SleB and the membrane-anchored protein YpeB, which influences germination through regulating SleB activity [93]. Although AOT13_08380 is not encoded alongside SleB/YpeB in the *P. thermoglucosidasius* DSM 2542 genome, homologues of both partner proteins are present (AOT13_15730; AOT13_15725).

Another *B. subtilis* BLP with a ring-building motif, GerM [94], has a BLP homologue, AOT13_17765 (58% identity). GerM likely functions as an accessory protein to the SpoIIIAH–SpoIIQ complex that bridges between the mother cell and forespore membranes during spore engulfment, with GerM–SpoIIIAH located in the mother cell membrane [94,95].

Finally, AOT13_04400 is a homologue of *B. subtilis* SsdC (YdcC), a σE-regulated BLP that influences spore shape and cortex formation, the latter in turn likely the cause of a germination delay in Δ*ssdC* mutant spores [96]. *B. subtilis* SsdC is a ring-forming protein apparently localized to the mother cell membrane near the forespore ‘mother cell proximal’ pole following forespore engulfment. How SsdC interacts across the mother cell-forespore junction remains unclear.

### BLP of unknown function

Fifteen BLPs have unknown functions. Of these, 11 cannot yet be confidently matched to conserved domains or protein families. However, AOT13_15640 (YphF) may have a function related to sporulation, as *yphF* in *B. subtilis* has been identified as a novel sporulation-related gene: deletion mutants produced spores with reduced heat resistance and aberrant morphology, due to inner spore coat defects [97]. Changes in the expression of genes affecting spore coat formation were also observed, including *spoIVA*. In both *B. subtilis* and *P. thermoglucosidasius*, *yphF* is encoded between *spoIVA* and a gene encoding a small DUF2768 integral membrane protein (YphE). Of the other ten BLPs unaffiliated to protein families, BCV53_19705 (annotated in strain NCIMB 11955) is encoded on plasmid pNCI002 and was not identified in either the DSM 2542 or strain Y4.1MC1 datasets, suggesting the absence of plasmid sequence data for those strains.

Four BLPs belong to identifiable protein families but cannot be confidently assigned a function. AOT13_04265 belongs to the terpenoid cyclase family and is noted to be encoded adjacent to another BLP of unknown function, AOT13_04270. AOT13_04770 (YerB) is a DUF3048 domain-containing protein and AOT13_14730 is a PCYCGC domain-containing BLP. The latter is encoded adjacent to AOT13_14725, a Fe^3+^ SBP (PF01497), and contains a conserved PCYCGC sequence (PF13798); aside from the three cysteines in the PCYCGC motif, four other cysteines are conserved in these proteins, one of which is the lipobox cysteine. AOT13_08275 is annotated as a cell-wall-binding lipoprotein (YkyA, PF10368), likely due to a corresponding low significance match to COG4942 for EnvC proteins; the latter regulate peptidoglycan hydrolases during cell division and contain an N-terminal coiled-coil domain that interacts with septal FtsEX [98,99]. The predicted structure of AOT13_08275 also suggests a coiled-coil structure, and so the interactions of this protein may be of interest. *B. subtilis* YkyA is apparently regulated in response to oxygen concentration [100].

### Comparison with *B. subtilis*

Fifty-seven of the *P. thermoglucosidasius* BLPs were identified as having a significant homologue in *B. subtilis* strain 168 (38 at >40% sequence identity), with matches to 46 proteins (File S4). Notably, of the 19 sequences matching proteins at <40% identity, 15 were SBP and 2 were germinant receptors, suggesting different substrate binding profiles. Of the 32 *P*. *thermoglucosidasius* BLPs without significant matches, it was notable that 11 were SBP and 13 (41%) were proteins for which functions could not be predicted. This latter may reflect a lack of knowledge of the mechanisms by which *P. thermoglucosidasius* has adapted to a thermophilic lifestyle.

### Proteomic analysis of BLP expression

BLPs were enriched in whole cell extracts using the Triton X-114 detergent extraction method, although it was noted that nearly 1,000 proteins were confidently detected (at least two peptides) in total, including many cytoplasmic and membrane proteins, indicating limited selectivity of the detergent extraction process. Expression of 45 BLPs was confidently detected (3 peptide matches in 3 replicate samples) by proteomic analysis, and a further 7 BLPs were detected with lower confidence (2 peptide matches), i.e. production of nearly 60% (52 out of 89) of the predicted lipoproteome was observed (File S6). Notably, none of the ten BLPs with sporulation-related functions were detected, consistent with the harvesting of cells during exponential phase. However, AOT13_15640 (YphF), tentatively linked to sporulation (see above), was detected. Reflecting the overall BLP profile, the majority [32] of proteins detected were SBP, notably those for amino acids/peptides (14 out of 18) and Fe-related (5 out of 5). SBP from all substrate categories was detected (Table 1), although only one of the five anion transport system SBPs (PF09084/PF13379).

Proteomic analysis also detected the expression of three predicted BLPs annotated as pseudogenes in the DSM 2542 genome and so not recovered in the initial bioinformatic analysis. AOT13_18640 (BCV53_18700) is annotated as encoded by a pseudogene, as the protein is truncated after 437 amino acids compared to the 690-amino-acid sequence encoded in strain Y4.1MC1 (GY4MC1_0728; File S1). Thus, the N-terminal part of this protein (100% identity to GY4MC1_0728 over 434 amino acids) is expressed despite lacking identifiable functional domains and lacking the putative glycosidase-like domain in the C-terminus of GY4MC1_0728.

Nine peptides were identified as matching the AOT13_09615 (BCV53_09625) pseudogene product, which is truncated after 140 amino acids, compared to the allelic GY4MC1_2570 in strain Y4.1MC1 (330 amino acids; File S1), which encodes a PF09084 SBP as part of a complete ABC locus. The cognate ATPase and permease proteins are retained in the type strain genomes (AOT13_09610 and AOT13_09620, respectively). Likewise, four peptides were also identified from AOT13_13205 (BCV53_13215), which is annotated as a 65-amino-acid sequence. Reinspection of the DSM 2542 genome shows that this sequence is the C-terminal part of a longer 131-amino-acid sequence, including a BLP signal peptide (as annotated in other *P. thermoglucosidasius* genomes such as strain 23.6, ORF IMI45_10385) and a domain homologous to an *Escherichia coli* toxin-immunity protein (PDB 8EY4_A).

## Conclusions

This study provides an inventory of BLP encoded in the *P. thermoglucosidasius* DSM 2542 type strain genome. These BLPs represent ~2.3% of the *P. thermoglucosidasius* reference proteome; moreover, given the number of potential interaction partners identified in the functional analysis (for example, with ABC transporters and TCS), it is evident that BLPs contribute to pathways involving 5–10% of the proteome. Nevertheless, it remains a limitation of the study that this is solely based on bioinformatic prediction, and experimental studies are needed to verify both the lipidation and the precise functions of the BLP identified here. Proteomic analysis detected the expression of ~66% (51 out of 79) of the non-sporulation-related BLP. Identification of the recently described LhaT biosynthetic pathway suggests that at least some of the BLP in *P. thermoglucosidasius* are post-translationally N-acetylated. These data contribute to understanding of the *P. thermoglucosidasius* cell envelope and how BLP may influence interactions of this bacterium with its environment.

## Abbreviations

BLPs: bacterial lipoproteins
PDB: Protein Data Bank
SBP: substrate-binding protein
TCS: two-component signalling system
